# Genetic diversity and inter-relationships of fruit bio-chemicals and antioxidant activity in Iranian wild blackberry species

**DOI:** 10.1038/s41598-020-75849-1

**Published:** 2020-11-04

**Authors:** Mehdi Garazhian, Ali Gharaghani, Saeid Eshghi

**Affiliations:** Department of Horticultural Science, School of Agriculture, Shiraz University, Shiraz, Iran

**Keywords:** Genetic variation, Plant hybridization

## Abstract

Blackberries are a rich source of bio-chemicals such as anthocyanins and polyphenolic antioxidants. The evaluation of the extent of variation among wild genetic resources can provide useful information for the establishment of effective conservation strategies and future breeding programs. In this study, variations and inter-relationship of berry weight, total phenol (TP), total soluble solids (TSS), titratable acidity (TA), ascorbic acid (AA), anthocyanin and antioxidant activity were estimated in their values among 57 accessions belonging to 4 different *Rubus* spp. native to Iran. The experiments were performed during two consecutive years (2014 and 2015). Combine analysis showed that there is no significant difference between the data of 2 years in all measured traits. High or very high levels of variations were detected in berry weight (0.14–1.30 g), antioxidant activity (40.21–88.08%), anthocyanin (80.74–145.09 mg/100 g), TSS (7.9–17.8 °Brix), TA (0.36–0.83%) and AA (9.56–20.92 mg/100 g). This is while TP showed very low levels of variation (109.5–129.1 mg/100 g). Correlation analysis showed that antioxidant activity correlated highly and positively with all of the measured characteristics including ascorbic acid (r = 0.927), anthocyanin (r = 0.752), total phenol (r = 0.681), TSS (r = 0.473) and berry weight (r = 0.541) except for TA. Cluster analysis based on all measured characteristics showed a partial differentiation between the accessions based on their species and, to lesser extent, according to their origin within the main clusters. Based on the bi-plot of the first two PCAs, genotypes and traits classified into four quadrants. This grouping was in agreement with that of cluster analysis, considering the fact that almost all of accessions in quadrants II and III (with a few exceptions) were same to those in the first clade of cluster analysis and the genotypes grouped in quadrants I and IV, represents the accessions of clade 2 in cluster analysis. The projection of the traits vectors in PCA were also fully in accordance to that of correlation analysis in almost all of studied traits. Results showed that a rich source of variations is available considering berry weight, fruit bio-chemical and antioxidant activity in the Iranian wild *Rubus* species, which needs immediate conservation and worth to be utilized in commercial breeding programs.

## Introduction

Blackberries belong to the Rosaceae family and the Rosoideae subfamily. Its genus is *Rubus* which is divided into 12 subgenera, with an estimated number of 750 species distributed worldwide^1^. The three largest subgenera of *Rubus* include *Idaeobatus, Malachobatus* and *Rubus*^2^. Among the *Rubus* genus, blackberries and raspberries are important fruit crops which are mostly produced as fresh fruits, processed food, frozen, juice, concentrate, jams, jellies or ice creams^3^. Raspberries and blackberries have a relatively short history of less than a century as cultivated crops and only a few of its generations have been collected from their wild progenitor species^4^. Fruits of blackberries are rich in anthocyanins and phenolic compounds that contribute to their high antioxidant capacity; therefore, the consumers’ interest is growing for blackberries because of their beneficial implications on human health^5,6^. The information on worldwide production, regions and quantity are not well known, even though blackberry production is rapidly increasing^7,8^ with an estimated 140,292 MT commercially harvested from 20,035 ha in 2005. Europe leads the world in its area under cultivation (7692 ha), while North America has the highest production (59,123 MT).

Restricted genetic diversity is a serious concern for future *Rubus* breeding programs, especially when selecting genotypes with superior quality fruit for processing and high yield. On the other hand, very little information is available on diversity in blackberry species, and few accessions are currently being utilized in blackberry breeding programs^9^. The evaluation of genetic and phenotypic diversity among wild and cultivated plant populations is important for the conservation of genetic resources, the identification of superior genotypes to be used as parents in breeding programs and for expanding the genetic background of blackberries^10^. There are various techniques available for the evaluation of plant phenotypic and genetic variability including morphological, bio-chemical and molecular markers. Morphological traits are the first to be used in the classification and evaluation of the germplasm^11–13^. In a report, Badjakov et al*.*^14^ analyzed 28 raspberry genotypes from the Bulgarian germplasm collection including 18 Bulgarian genotypes and breeding lines, 8 accessions from outside Bulgaria and two accessions of wild species, including *R. occidentalis* and *R. adiene*. The genotypes they studied were clearly clustered into two groups, corresponding to two pedigree groups. The investigation of genetic diversity of *R. alceifolius* in its native range in Southeast Asia as well as its introduced range in Madagascar and the neighboring parts of the Indian Ocean islands have confirmed greater genetic diversity in its native range^15^. Furthermore, Gharaghani et al.^16^ investigated the diversity of fruit quantity and chemical properties of *R. sanctus* Schreb. genotypes from two distinct regions of northern and southern Iran. They reported high variability as well as considerable differences in fruit characteristics between the genotypes collected from the two different parts of Iran.

There is no documented data available about the cultivation of *Rubus* spp. crops in Iran; however, the collection of blackberry fruits from wild stands is a common activity among Iranian locals of the Caspian region, and the sale of blackberries is a frequent sight along the roadsides of northern Iran. Based on a recent survey, *Rubus* genetic resources comprise 8 species in Iran including *Rubus saxatilis* L. (the only herbaceous type that belongs to the *Cylactis* Subgenus), *R. caesius* L., *R. sanctus* Schreber (syn. *R. anatolicus* (Focke) Hausskn.), *R. hirtus* Waldst. & Kit. (syn. *R. lanuginosus* Stev. ex Ser)*, R. hyrcanus* Juz.*, R. dolichocarpus* Juz. (syn. *R. ochtodes* Juz.)*, R. discolor* Weihe and Nees (syn. *R. armeniacus* Focke) and *R. persicus* Boiss (syn. *R. raddeanus* Focke). All of these species belong to the subgenus *Rubus,* except for *R. saxatilis* L., the only herbaceous type, that belongs to the *Cylactis* Subgenus. Five inter-specific natural hybrids also have been reported from Iran^17^. However, apart from the reports on botanical studies, there is little information on pomology and nutritional values of the available wild *Rubus* genetic resources in Iran. Accordingly, the objectives of this study were to describe the variability of berry size, fruit bio-chemical characteristics and antioxidant activities in 57 genotypes of the Iranian wild blackberry, representing four species. Objectives also dealt with discovering useful correlations among the measured traits and determining the relationships between the genotypes and species by using cluster analysis. These were performed to provide necessary information on the domestication and breeding of this valuable crop.

## Material and methods

### Plant material

**Figure 1:**
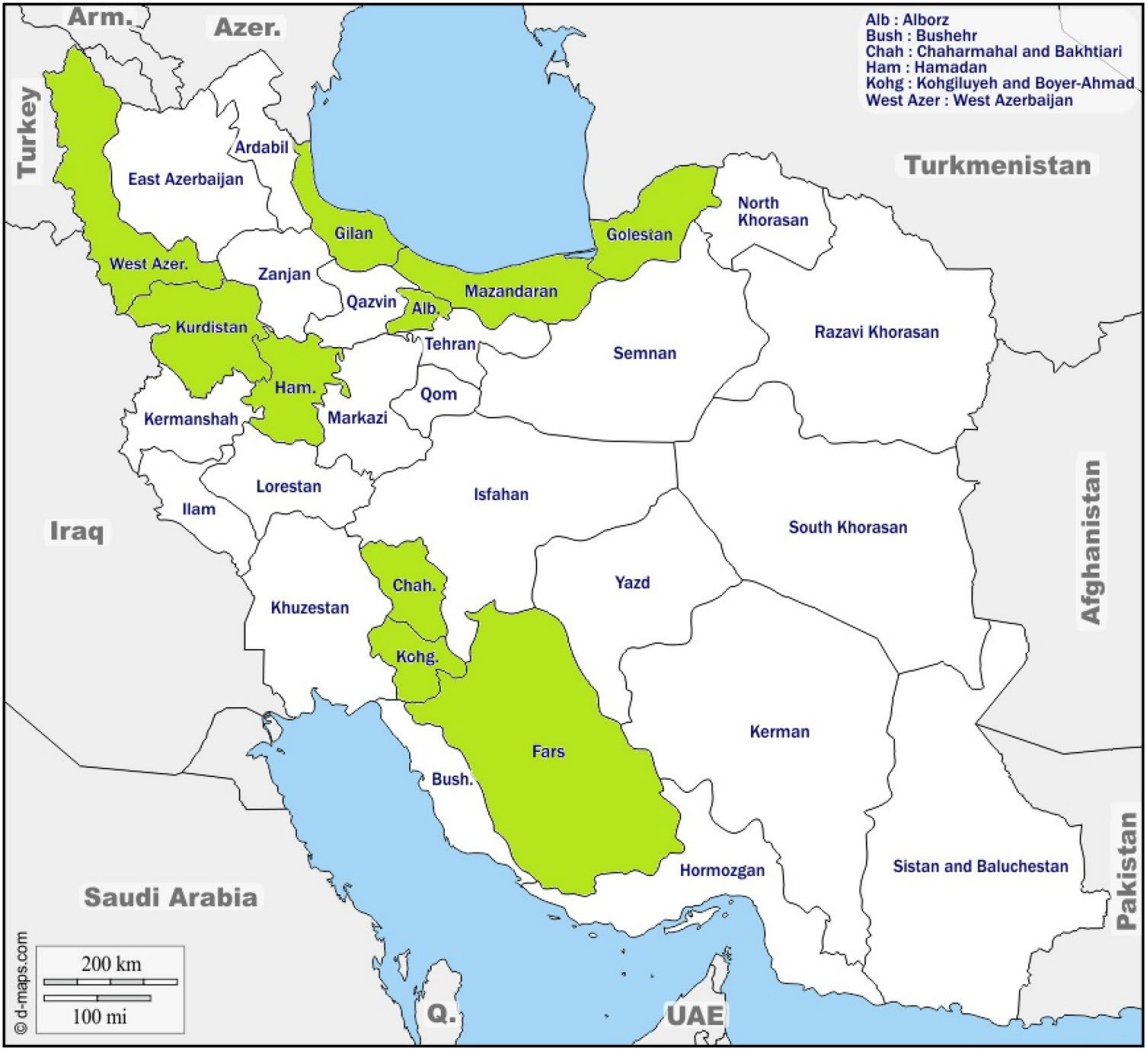
Approximate distribution of blackberry habitats in Iran that used in our study (the green colored provinces). The original map is obtained from d-map (https://d-maps.com/carte.php?num_car=5496&lang=en) and modified (colored) using paint software of Microsoft Windows 10).

In this study, fruit samples were collected from first Iranian blackberry repository, established in 2010 at Shiraz University, Iran^18^. The geographic coordination of the established collection comprised a longitude of 52° 35′ 0.73′′, a latitude of 29° 43′ 43.56′′ and an altitude of 1791.4 (m). The average long-term annual rainfall was 386 mm, and the soil was silty-loam. To establish the repository genotypes which included four different species, *R. sanctus, R. hirtus, R. caesius* and *R. persicus,* were collected from 10 provinces across Iran (Fig. 1). The geographic information of the origin as well as the species of the genotypes under study is presented in Table 1.

**Table 1:** Geographic information of original sites in different parts of Iran, in which 57 studied blackberry genotypes, collected from.

Species	Genotype (site) name	Province	E (longitude)	N (latitude)	Altitude
*R. sanctus*	Kazerun (Fathabad)	Fars	51° 31′ 21.72′′	29° 43′ 42.59′′	755.2 m
	Kazerun (Eslamabad)	Fars	51° 34′ 26.50′′	29° 46′ 58.43′′	797.3 m
	Nourabad (Bavan)	Fars	51° 32′ 22.98′′	30° 07′ 36.14′′	1079.9 m
	Dasht Arzhan	Fars	51° 58′ 46.66′′	29° 39′ 10.06′′	2142.1 m
	Sepidan (Roodbal)	Fars	52° 02′ 20.75′′	30° 06′ 00.10′′	1920.2 m
	Beyza (Hoseinabad)	Fars	52° 23′ 24.53′′	29° 58′ 14.28′′	1637.0 m
	Kamfiruz	Fars	52° 11′ 32.76′′	30° 19′ 35.13′′	1783.0 m
	Firouzabad	Fars	52° 32′ 21.76′′	28° 52′ 37.76′′	1347.2 m
	Jahrom (Khafr)	Fars	53° 31′ 31.93′′	28° 29′ 15.77′′	1049.4 m
	Sivand	Fars	52° 55′ 01.58′′	30° 05′ 12.83′′	1802.8 m
	Seyedan (Bag Bonyad)	Fars	52° 35′ 03.47′′	30° 57′ 03.11′′	1689.4 m
	Shiraz (Chamran)	Fars	52° 29′ 41.80′′	29° 39′ 12.50′′	1674.5 m
	Dena (Karyak)	Kohgiluyeh-Boyrahmad	51° 25′ 05.97′′	30° 49′ 01.80′′	2018.9 m
	Kakan	Kohgiluyeh-Boyrahmad	51° 48′ 03.78′′	30° 37′ 31.62′′	2013.2 m
	Yasuj (Naregah)	Kohgiluyeh-Boyrahmad	51° 34′ 08.08′′	30° 36′ 55.97′′	2105.2 m
	Jade Kandovan	Alborz	46° 37′ 36.78′′	37° 59′ 25.16′′	801.0 m
	Urmia 1	West Azarbayejan	44° 54′ 20.88′′	37° 25′ 56.32′′	1794.6 m
	Urmia 2	West Azarbayejan	44° 55′ 30.15′′	37° 39′ 34.54′′	2032.1 m
	Sanandaj 1	Kurdistan	47° 03′ 04.14′′	35° 13′ 01.75′′	1588.6 m
	Abidar	Kurdistan	50° 32′ 40.61′′	36° 2′ 25.56′′	1890.6 m
	Malayer	Hamedan	48° 50′ 41.89′′	34° 14′ 03.40′′	1862.6 m
	Roodbar	Guilan	49° 27′ 02.04′′	36° 48′ 10.00′′	1639.5 m
	Kelachay	Guilan	50° 22′ 53.58′′	37° 00′ 22.53′′	1804.1 m
	Roodsar	Guilan	50° 16′ 33.97′′	37° 08′ 24.95′′	1630.3 m
	Kelachay (Polrood)	Guilan	50° 22′ 29.17′′	37° 04′ 40.88′′	1680.0 m
	Talesh 2	Guilan	48° 52′06.85′′	37° 48′ 40.60′′	766.8 m
	Astara 4	Guilan	48° 46′24.82′′	38° 25′ 21.13′′	531.2 m
	Gardane Heyran 1	Guilan	46° 57′04.84′′	38° 49′ 20.26′′	645.8 m
	Gardane Heyran 4	Guilan	46° 47′37.52′′	38° 47′ 13.90′′	776.5 m
	Sari1(Jade Khazar)	Mazandaran	53° 01′08.24′′	36° 40′ 08.83′′	544.6 m
	Jade Haraz	Mazandaran	52° 17′17.34′′	35° 56′ 42.56′′	403.5 m
	Chaloos	Mazandaran	51° 26′04.23′′	36° 38′ 00.01′′	1547.7 m
	Tonekabon (Nematabad)	Mazandaran	50° 55′11.70′′	36° 45′ 09.33′′	1821.7 m
	Namak Abrud 2	Mazandaran	51° 20′47.38′′	36° 38′ 24.21′′	1625.1 m
	Namak Abrud 3	Mazandaran	51° 15′43.71′′	36° 39′ 08.39′′	1376.4 m
	Babolsar 1	Mazandaran	52° 45′29.09′′	36° 38′ 26.80′′	− 22.2 m
	Babolsar 2	Mazandaran	52° 34′12.71′′	36° 37′ 42.13′′	− 24.6 m
	Sari 3	Mazandaran	53° 03′ 21.17′′	36° 34′ 48.79′′	96.1 m
	Behshahr 2	Mazandaran	53° 34′ 22.62′′	36° 40′ 41.79′′	359.0 m
	Bandar Gaz	Golestan	53° 56′ 13.67′′	36° 46′ 28.74′′	409.0 m
	Gorgan 2	Golestan	54° 25′ 58.79′′	36° 50′ 35.75′′	501.3 m
	Naharkhoran	Golestan	54° 27′ 45.60′′	36° 47′ 2.595′′	413.4 m
*R. hirttus*	Ganjname	Hamedan	48° 26′ 06.01′′	34° 45′ 34.13′′	1795.2 m
	Gerdbisheh	Char Mahal-Bakhtiari	50° 39′ 37.12′′	32° 8′ 12.91′′	2062.8 m
	Ashkvarat	Guilan	50° 16′ 44.19′′	36° 49′ 39.56′′	1868.1 m
	Ashkvarat 1	Guilan	50° 16′ 70.76′′	36° 48′ 05.24′′	1835.2 m
	Ashkvarat 2	Guilan	50° 14′ 70.40′′	36° 46′ 26.55′′	1839.9 m
	Anzali 2	Guilan	49° 28′ 44.39′′	37° 27′ 12.35′′	1173.7 m
	Rezvanshahr	Guilan	49° 08′ 32.03′′	37° 33′ 33.37′′	950.3 m
	Astara 1	Guilan	48° 46′ 45.47′′	38° 26′ 56.21′′	490.4 m
	Astara 2	Guilan	48° 47′ 43.30′′	38° 22′ 49.72′′	558.6 m
	Abas Abad	Mazandaran	51° 07′ 24.50′′	36° 42′ 34.78′′	1722.7 m
*R. caesius*	Aliabad Katul 1	Golestan	54° 54′ 45.18′′	36° 54′ 54.92′′	150.4 m
	Aliabad Katul 2	Golestan	54° 51′ 2.03′′	36° 53′ 32.20′′	159.7 m
	Gorgan 5	Golestan	54° 26′ 21.73′′	36° 50′ 44.32′′	499.8 m
	Fuman	Guilan	49° 18′ 41.58′′	37° 12′ 45.17′′	1325.5 m
*R. persicus*	Masule	Guilan	49° 00′ 2.22′′	37° 09′ 40.34′′	1231.3 m

This research was conducted in two growing seasons during 2014 and 2015, on 5-year-old canes propagated on its own root at 4 × 2 m spacing and a free training system. A total of 57 wild genotypes out of more than 100, were selected and tagged as experimental plant materials. Each genotypes represents three cane in the repository, which treated as three replicate. The orchard floor was managed using mechanical cultivation between rows and hand cultivation within the rows. Sever pruning (including removal of all canes from 30 cm above ground) were done manually in all of the experimental plants on early March. No fruitlet thinning was applied. Canes were irrigated using a drip irrigation system with 3-day intervals and the amount of irrigation water was adjusted based on the plants’ requirement during the growing season.

The regular nutrition program of the orchard consisted of an application of 20 t ha^–1^ of decomposed dairy cattle manure, 200 kg ha^–1^ of Di-Ammonium phosphate, 200 kg ha^–1^ potassium sulfate every other year, and also 100 kg of Ammonium sulfate annually divided in two applications of equal amounts in autumn (early November) and spring (mid-May) using a fertigation system; plus, one foliar spray of a mix of trace elements in early June. No chemical spry used for plant protection.

Fruits of each genotypes were sampled at its appropriate ripening time (Fully black color stage) and immediately transferred to the lab for further evaluation.

### Determination of fruit physico-chemical properties

Fruit samples were harvested in their ripe stage (black color) and fruit characteristics including fresh weight, total soluble solid (TSS), titratable acidity (TA), total phenolic compound, ascorbic acid, total anthocyanin content and antioxidant activity were evaluated. Samples were in three replicates. In order to measure the berry weight, a sample of 20 fruits per replicate was randomly selected and weighed by using an electronic balance with 0.001 g precision, and then the weight was averaged and reported as g per berry. TSS was determined by using a manual refractometer (ATAGO, Japan). TA content was measured as citric acid equivalent by diluting 5 ml aliquot of fruits juice in 40 ml of distilled water, which was then titrated to pH 8.2 by using 0.1 N NaOH, and was finally expressed as g/100 g of fresh weight, according to descriptions by Zheng et al.^19^. The total phenolic compound was determined by using a spectrophotometer (Epoch microplate spectrophotometer, USA) based on the Folin–Ciocalteu colorimetric method^20^. For this purpose, the fruit extract (1 ml) was mixed with 1 ml of HCl (6 mol) and 5 cc of methanol (75%). Then, 1 cc of the solution was mixed with 5 ml of 10% Folin-Ciocalteu reagent and 15 ml of NaCO_3_ $\left(\frac{7\mathrm{g}}{100\mathrm{ ml}}\right)$. It was then tested by spectrophotometry at 760 nm. The results were expressed as mg Gallic acid/100 g fresh weight. Gallic acid was used as a standard compound and the total phenols were expressed as mg/g Gallic acid equivalent by using the standard curve equation: y = 0.0061x + 0.0396, R^2^ = 0.9991, where x is the absorbance at 760 nm and y is total phenolic content in the different extracts.

Ascorbic acid was quantitatively determined according to 2, 6-dichlorophenol-indophenol (DCPIP) dye method^21^. The ascorbic acid in 0.1 g of fresh fruit was extracted by using 2% (w/v) m-phosphoric acid. The extract volume was increased to reach 100 ml, and then 1 cc of the solution was mixed with 9 cc of 2, 6-dichlorophenol-indophenol (50 μmol). The absorbance of the solution was measured at 515 nm by using the spectrophotometer (Epoch microplate spectrophotometer, USA). Ascorbic acid content was calculated according to the following formula: Ascorbic acid [mg/100 g] = Corrected Volume × F × 40, (F = M/g of Ascorbic acid equipollent of ml indophenol).

Total anthocyanin content of fruit extract was estimated by using the pH differential method proposed by AOAC International^22^. Absorbance was measured with the spectrophotometer at 510 and 700 nm, at the pH value of 1 and 4.5, where A = (A510–A700) pH 1 and (A510–A700) pH 4.5. The data were calculated by using the extinction coefficient for cyanidin-3-glucoside and were expressed as mg cyanidin 100 g^−1^ fresh weight.

The antioxidant activity was evaluated by using the 1, 1-dipheny l-2-picrylhydrazyl (DPPH) free radical scavenging method. The DPPH free radical scavenging activity measurements were carried out according to the method of Ao et al.^23^. Briefly, 50 μl of the fruit extracts were added to 950 μl of DPPH radical and were vortexed for 30 s. They were kept at room temperature in darkness for 15 min. The absorbance of the samples was measured at 515 nm by using the spectrophotometer (Epoch microplate spectrophotometer, USA). For each sample, three separate determinations were carried out. The antioxidant activity was expressed as the percentage of decline in absorbance, compared to that of the control, and corresponding to the percentage of scavenged DPPH. The percentage of scavenged (% DPPHsc) was calculated by using the following formula: % DPPHsc = (A cont − A samp) × 100/A cont, where ‘A cont’ is the absorbance of the control, and ‘A samp’ is the absorbance of the sample.

### Statistical analysis

Data were analyzed on the basis of a randomly complete block design (RCBD) with three replicates. Analysis of variance were carried out by SAS 9.1 software. Initially, the data were checked for differences between the two growing seasons. There were no significant differences between the 2 years and the interaction between year and genotype was not statistically significant (Table 2). Therefore, data pertaining to the 2 years were averaged and accordingly used for further analysis. Diversity index were determined as indicators of variability which was calculated using this formula^24^; Diversity Index = (Std. dev./Mean) × 100, Std. dev. = Std. error × √n, n = 57. The correlations between all morphological variables studied herein as well as between these variables and geographic data of original sites in which plant materials were sampled, calculated based on Pearson’s Simple Correlation Coefficient by using the SPSS software (version 17). Principal component analysis (PCA) was performed and first two components were used to create bi-plot illustrating the relationships of the accessions as well as the measured traits. Cluster analysis of the accessions was performed by incorporating all of the measured attributes into the analysis. This was carried out according to Euclidian distance measure based on Wards method by the R software. The different variables were scaled based on Z-score prior to cluster analysis. The graph that presents the classification is a dendrogram of dissimilitude with standardized Euclidean Distances as they group together the closest accessions in homogeneous groups.

**Table 2:** Analysis of variance of studied traits in 57 blackberry genotypes assigned to four wild species.

Sources of variations	Degree of freedom	Mean-square						
		Berry weight	Antioxidant activity	Anthocyanin	TSS	Total phenol	Titrateable acid	Ascorbic acid
Year	1	0.06^ns^	4.10^ns^	14.49^ns^	4.06^ns^	27.69^ns^	0.00^ns^	13.92^ns^
Error	4	0.001	40.06	43.72	2.03	11.17	0.003	3.01
Genotype	56	0.3**	1027.19**	3247.82**	35.54**	200.79**	0.08**	48.53**
Year × Genotype	56	0.001^n.s^	3.4^ns^	4.13^ns^	0.37^ns^	3.72^ns^	0.00^ns^	0.46^ns^
Error	224	0.0009	3.45	6.23	0.75	3.58	0.000	0.61
CV (%)	–	3.65	2.85	2.12	7.41	1.57	5.37	4.87

*CV* Coefficient of variation.

^ns^ and ** represent non significance and significance at the 1% level respectively.

## Results and discussion

### Diversity of fruit characteristics

#### Berry weight

Fresh berry weight of all accessions ranged from 0.14 to 1.30 g. ‘Sepidan’ (Roodbal) and ‘Babolsar 2′ accessions belong to the *R. sanctus*, had the largest and smallest berry sizes respectively (Table 3). In general, a high level of variation (diversity index = 30.22) was recorded for berry size among the accessions and species studied (Table 4). Yilmaz et al.^6^ investigated the fruit weight of 9 cultivated blackberries and 16 blackberry genotypes collected from the wild *(Rubus fruticosus* L.) in Turkey. A range of 0.4–1.2 g of berry weight was reported for the wild genotypes and 1.2–5.4 g for the commercial cultivars. The range, as mentioned for the wild genotypes, is consistent but less broad than the range of berry weight reported in this study (0.14 to 1.30 g). On the other hand, Celik et al.^25^ reported a higher range of fruit weight (1.5–2.1 g) for wild blackberries in Turkey. These differences could arise from different species, varied numbers of the accessions under each case of study, as well as the diverse climatic conditions in each context^11,26,30^. Results of correlation analysis showed that berry weight has a significant, but negative correlation (r =  − 0.287) with latitude (Table 5), illustrating the effect of climatic and geographic parameters of the original sites in which the plant materials were collected.

**Table 3:** Variations of berry weight, fruit bio-chemicals and antioxidant activity in 57 blackberry genotypes assigned to four wild species (mean of 2014 and 2015).

Species	Genotype	Berry weight(g) ± SD	Antioxidant activity(%) ± SD	Anthocyanin (mg/100 g) ± SD	TSS (°Brix) ± SD	Total phenol (mg/100 g) ± SD	Titrateable acid(%) ± SD	Ascorbic acid (mg/100 g) ± SD
*R. sanctus*	Kazerun (Fathabad)	1.24 ± 0.04	70.79 ± 1.71	134.68 ± 3.80	12.00 ± 1.20	117.8 ± 3.49	0.52 ± 0.04	15.99 ± 1.13
	Kazerun (Eslamabad)	0.99 ± 0.02	74.16 ± 1.78	143.49 ± 2.37	17.80 ± 0.93	128.2 ± 1.97	0.64 ± 0.03	18.76 ± 0.63
	Nourabad (Bavan)	0.73 ± 0.03	40.21 ± 1.28	105.86 ± 4.79	14.00 ± 0.89	114.1 ± 1.94	0.50 ± 0.02	09.56 ± 0.76
	Dasht Arzhan	0.69 ± 0.03	40.94 ± 1.39	108.79 ± 3.45	14.20 ± 0.75	115.0 ± 1.67	0.63 ± 0.02	10.28 ± 0.83
	Sepidan (Roodbal)	1.30 ± 0.06	61.68 ± 5.56	137.11 ± 2.57	12.50 ± 1.10	109.5 ± 2.26	0.44 ± 0.02	16.74 ± 1.13
	Beyza (Hoseinabad)	1.21 ± 0.03	71.96 ± 3.46	142.88 ± 3.97	12.58 ± 0.66	112.5 ± 1.52	0.53 ± 0.02	17.69 ± 0.58
	Kamfiruz	0.39 ± 0.02	66.02 ± 1.46	121.16 ± 2.72	14.25 ± 0.75	118.0 ± 3.08	0.62 ± 0.03	16.97 ± 0.97
	Firouzabad	1.13 ± 0.04	72.55 ± 1.78	140.14 ± 2.86	12.90 ± 1.10	116.6 ± 1.86	0.43 ± 0.02	17.94 ± 0.51
	Jahrom (Khafr)	1.00 ± 0.03	77.41 ± 1.90	140.16 ± 3.27	12.50 ± 1.48	119.5 ± 1.87	0.41 ± 0.01	17.45 ± 1.18
	Sivand	1.06 ± 0.06	72.94 ± 2.16	130.08 ± 2.32	12.08 ± 0.60	124.0 ± 1.41	0.52 ± 0.04	17.72 ± 0.97
	Seyedan (Bag Bonyad)	0.91 ± 0.02	72.08 ± 1.87	128.43 ± 1.47	10.08 ± 0.90	116.5 ± 1.38	0.44 ± 0.02	18.12 ± 0.38
	Shiraz (Chamran)	0.83 ± 0.02	74.05 ± 2.05	140.13 ± 1.75	11.70 ± 0.80	119.5 ± 1.05	0.67 ± 0.02	16.76 ± 1.26
	Dena (Karyak)	0.79 ± 0.02	79.88 ± 2.42	136.33 ± 1.50	12.40 ± 0.49	122.0 ± 1.67	0.63 ± 0.02	17.25 ± 1.10
	Kakan	0.89 ± 0.02	79.62 ± 1.34	134.76 ± 1.81	13.60 ± 0.50	124.5 ± 1.87	0.73 ± 0.03	17.60 ± 0.69
	Yasuj (Naregah)	0.89 ± 0.02	88.08 ± 1.38	143.82 ± 2.51	14.90 ± 0.60	110.3 ± 1.75	0.42 ± 0.03	20.65 ± 0.56
	Jade Kandovan	0.79 ± 0.02	44.00 ± 2.20	086.46 ± 2.08	08.08 ± 0.66	116.0 ± 1.90	0.36 ± 0.02	11.72 ± 0.66
	Urmia 1	0.90 ± 0.02	69.58 ± 1.47	110.80 ± 2.57	09.41 ± 0.91	116.0 ± 1.90	0.67 ± 0.02	16.50 ± 0.85
	Urmia 2	0.88 ± 0.03	79.23 ± 2.08	134.56 ± 2.49	15.20 ± 0.60	121.8 ± 2.32	0.72 ± 0.02	18.70 ± 0.74
	Sanandaj 1	0.92 ± 0.01	80.35 ± 1.97	141.08 ± 2.52	15.00 ± 0.66	125.3 ± 1.37	0.58 ± 0.03	19.38 ± 0.93
	Abidar	0.90 ± 0.02	84.22 ± 1.44	145.09 ± 2.10	14.20 ± 0.40	125.6 ± 1.51	0.63 ± 0.03	20.53 ± 0.79
	Malayer	0.57 ± 0.02	72.12 ± 1.54	134.91 ± 1.98	14.50 ± 0.66	125.6 ± 1.51	0.61 ± 0.02	18.21 ± 0.73
	Roodbar	1.02 ± 0.05	73.32 ± 1.27	140.17 ± 2.59	11.40 ± 0.60	122.3 ± 1.86	0.56 ± 0.02	17.80 ± 0.94
	Kelachay	0.81 ± 0.01	65.01 ± 1.64	130.95 ± 2.31	11.08 ± 0.91	121.8 ± 1.47	0.46 ± 0.02	16.27 ± 0.70
	Roodsar	0.88 ± 0.02	61.65 ± 2.15	124.38 ± 1.74	10.40 ± 1.10	125.8 ± 1.33	0.66 ± 0.02	15.51 ± 1.02
	Kelachay (Polrood)	0.87 ± 0.02	70.81 ± 1.76	130.49 ± 3.27	12.08 ± 0.66	124.0 ± 2.10	0.58 ± 0.02	17.25 ± 0.68
	Talesh 2	0.84 ± 0.02	47.55 ± 1.01	85.75 ± 02.47	07.90 ± 0.49	113.5 ± 1.87	0.43 ± 0.03	12.89 ± 0.61
	Astara 4	1.03 ± 0.04	72.58 ± 1.28	135.09 ± 2.23	11.58 ± 0.49	127.3 ± 1.51	0.52 ± 0.03	18.40 ± 0.36
	Gardane Heyran 1	1.16 ± 0.03	75.72 ± 1.35	135.03 ± 2.43	11.50 ± 0.80	125.8 ± 1.33	0.55 ± 0.03	19.00 ± 0.51
	Gardane Heyran 4	0.41 ± 0.02	50.43 ± 1.66	84.857 ± 2.25	08.33 ± 0.80	110.8 ± 1.72	0.60 ± 0.02	13.66 ± 1.18
	Sari1(Jade Khazar)	0.92 ± 0.02	74.16 ± 1.51	142.23 ± 3.51	14.40 ± 0.90	124.3 ± 1.97	0.43 ± 0.03	18.00 ± 0.54
	Jade Haraz	1.05 ± 0.04	76.55 ± 2.01	141.11 ± 1.67	14.70 ± 1.08	129.3 ± 1.75	0.42 ± 0.02	17.72 ± 0.53
	Chaloos	1.13 ± 0.06	65.23 ± 1.45	135.34 ± 1.54	11.41 ± 1.11	124.6 ± 1.63	0.45 ± 0.02	16.19 ± 0.91
	Tonekabon(Nematabad)	0.65 ± 0.01	64.14 ± 2.42	121.21 ± 1.68	13.40 ± 1.10	115.5 ± 1.64	0.55 ± 0.02	15.90 ± 0.58
	Namak Abrud 2	0.77 ± 0.02	52.38 ± 1.10	085.53 ± 1.93	08.50 ± 0.54	110.0 ± 2.28	0.83 ± 0.03	13.88 ± 0.76
	Namak Abrud 3	0.88 ± 0.02	55.96 ± 1.24	111.14 ± 2.79	12.30 ± 0.80	125.1 ± 1.94	0.69 ± 0.03	14.73 ± 0.34
	Babolsar 1	1.13 ± 0.04	80.14 ± 1.49	135.38 ± 1.60	12.08 ± 0.66	127.6 ± 2.42	0.52 ± 0.03	19.67 ± 0.85
	Babolsar 2	0.14 ± 0.02	61.23 ± 1.49	082.35 ± 2.75	10.33 ± 0.51	112.3 ± 1.37	0.62 ± 0.02	16.42 ± 0.95
	Sari 3	1.10 ± 0.07	84.48 ± 1.06	134.00 ± 2.41	09.50 ± 1.04	126.1 ± 1.47	0.42 ± 0.02	20.92 ± 0.80
	Behshahr 2	0.69 ± 0.04	79.56 ± 1.30	133.77 ± 2.33	09.66 ± 0.81	123.0 ± 2.37	0.61 ± 0.02	20.13 ± 0.95
	Bandar Gaz	0.37 ± 0.03	41.05 ± 1.26	085.10 ± 2.67	08.58 ± 0.49	116.3 ± 1.21	0.55 ± 0.03	11.84 ± 0.48
	Gorgan 2	0.44 ± 0.04	52.07 ± 1.55	090.51 ± 1.99	09.16 ± 0.75	113.8 ± 1.47	0.66 ± 0.03	13.39 ± 0.95
	Naharkhoran	0.70 ± 0.03	56.87 ± 1.63	133.70 ± 2.26	11.70 ± 1.00	115.2 ± 3.14	0.74 ± 0.05	11.09 ± 0.62
*R. hirttus*	Ganjname	0.90 ± 0.03	62.35 ± 2.88	081.45 ± 1.99	12.50 ± 1.00	115.9 ± 2.51	0.65 ± 0.03	14.90 ± 1.37
	Gerdbisheh	0.84 ± 0.03	55.35 ± 1.09	092.12 ± 1.59	09.16 ± 0.75	119.1 ± 2.32	0.47 ± 0.03	14.22 ± 0.61
	Ashkvarat	0.82 ± 0.01	60.72 ± 3.34	080.74 ± 3.02	15.30 ± 0.98	123.4 ± 3.85	0.68 ± 0.02	14.89 ± 0.72
	Ashkvarat 1	0.84 ± 0.02	50.33 ± 1.45	095.43 ± 1.88	09.58 ± 0.49	119.6 ± 1.86	0.41 ± 0.03	14.91 ± 0.62
	Ashkvarat 2	0.71 ± 0.02	45.87 ± 1.36	080.75 ± 2.54	08.75 ± 0.52	109.6 ± 1.97	0.53 ± 0.03	14.94 ± 0.62
	Anzali 2	0.89 ± 0.03	71.32 ± 1.45	140.67 ± 2.95	11.60 ± 0.81	129.1 ± 1.94	0.73 ± 0.02	18.07 ± 0.52
	Rezvanshahr	0.40 ± 0.01	45.36 ± 1.10	081.00 ± 2.51	08.20 ± 0.40	111.3 ± 2.34	0.40 ± 0.01	11.13 ± 0.48
	Astara 1	0.51 ± 0.02	48.34 ± 1.05	091.28 ± 2.45	08.91 ± 0.80	120.0 ± 1.26	0.79 ± 0.02	12.44 ± 0.79
	Astara 2	0.89 ± 0.03	71.04 ± 1.38	135.94 ± 2.09	11.41 ± 0.49	124.8 ± 1.17	0.63 ± 0.03	18.29 ± 0.87
	Abas Abad	0.41 ± 0.02	52.13 ± 1.36	094.25 ± 2.55	08.30 ± 0.81	112.1 ± 1.72	0.72 ± 0.03	14.00 ± 0.86
*R. caesius*	Aliabad Katul 1	0.40 ± 0.02	54.24 ± 1.44	083.36 ± 3.43	08.60 ± 0.91	114.6 ± 1.63	0.71 ± 0.02	14.55 ± 0.51
	Aliabad Katul 2	0.86 ± 0.05	81.49 ± 1.67	130.35 ± 1.98	12.10 ± 1.03	127.3 ± 1.63	0.79 ± 0.03	20.00 ± 0.98
	Gorgan 5	1.06 ± 0.05	59.66 ± 0.84	085.58 ± 1.73	09.00 ± 1.26	120.6 ± 1.51	0.51 ± 0.02	14.82 ± 0.62
	Fuman	0.45 ± 0.02	41.92 ± 2.38	100.60 ± 2.48	14.66 ± 0.98	117.6 ± 1.74	0.40 ± 0.04	10.03 ± 0.61
*R. persicus*	Masule	0.83 ± 0.02	73.00 ± 1.95	094.92 ± 2.52	16.40 ± 0.90	122.9 ± 2.26	0.68 ± 0.03	17.07 ± 1.39

**Table 4:** Descriptive Statistics of berry weight, fruit bio-chemical characteristic and antioxidant activity in 57 blackberry genotypes assigned to four wild species (mean of 2014 and 2015).

Traits	Mean	Std. error	Range	Max	Min	Std. dev	Diversity Index*
Weight (g)	0.825	0.023	1.218	1.360	0.141	0.249	30.27
Antioxidant (%)	65.02	1.22	49.28	89.15	39.87	13.05	20.07
Anthocyanin (mg/100 g)	117.67	2.17	66.37	146.18	79.81	23.18	19.70
TSS (°Brix)	11.748	0.228	10.667	18.333	7.667	2.440	20.77
Phenol (mg/100 g)	119.88	0.545	22.00	130.33	108.33	05.82	04.86
Titrateable acid (%)	0.5798	0.0109	0.4867	0.8500	0.3633	0.118	20.48
Ascorbic acid (mg/100 g)	16.135	0.267	12.193	21.593	9.400	2.853	17.68

*Diversity Index = (Std. dev./Mean) × 100, Std. dev. = Std. error × √n, n = 57.

**Table 5:** Simple Pearson correlations between berry weight and fruit bio-chemical characteristic with longitude, latitude and altitude of original site of 57 blackberry genotypes assigned to four wild species (mean of 2014 and 2015).

	Fruit weight (g)	Antioxidant activity (%)	Anthocyanin (mg/100 g)	TSS (°Brix)	Total phenol (mg/100 g)	Titrateable acid (%)	Ascorbic acid (mg/100 g)
E (longitude)	− 0.012^ns^	0.060^ns^	0.108^ns^	− 0.027^ns^	− 0.042^ns^	− 0.028^ns^	0.037^ns^
N (latitude)	− 0.287*	− 0.218^ns^	− 0.366**	− 0.387**	0.164^ns^	0.179^ ns^	− 0.118^ns^
Altitude (m)	0.127^ns^	0.036^ns^	0.110^ns^	0.287*	− 0.161^ns^	0.020^ ns^	0.010^ns^

*and **: Significant different at 5% and 1% probability levels respectively.

#### Antioxidant activity

As presented in Table 3, the antioxidant activity varied from 88.08% to 40.21% among the accessions studied. The genotypes ‘Yasuj’ (Naregah) and ‘Nourabad’ (Bavan), both of which belong to the *R. sanctus*, were recognized as accessions with the highest and lowest rates of antioxidant activity, respectively (Table 3). The evaluation of antioxidant activity among the genotypes of Iranian blackberry species in this study showed a relatively high level of variability (Diversity index = 20.07) (Table 4). Diversity in the antioxidant activity of blackberry can be attributed to the various methods of cultivation, environmental conditions during harvest and, especially, genetic differences^26,27^. Although we didn’t find any significant association in correlation analysis between geographic parameters of the original habitats of used plant materials and measured values for antioxidant activity (Table 5), but according to Wang^28^, the growth of blackberry through cold nights and cool days can reduce the antioxidant capacity of fruits. However, it should be noted that organisms causing disease and pests can have an impact on the plants phenolic compounds synthesis, which could influence antioxidant activity. Gundogdu et al.^11^ reported the range of 30.855–48.900 μmol TE g^–1^ (TE = Trolox equivalent antioxidant capacity) for antioxidant capacity in blackberry cultivars from East Anatolia. Huang et al*.*^29^ estimated the antioxidant activity of blackberry compared to blueberry and strawberry, whereby it was proved that blueberry had the highest antioxidant capacity (14.98 mmol Trolox/100 g DW) followed by blackberry (11.48 mmol Trolox/100 g DW) and strawberry (4.44 mmol Trolox/100 g DW). Those results were in line with the results of the current study, however the methods used for measurements were different.

#### Anthocyanin

The fruits’ anthocyanin content in all accessions belonging to the four species ranged from 80.74 to 145.09 mg/100 g. Herein, the ‘Abidar’ (*R. sanctus*) and ‘Ashkvarat’ (*R. hirtus*) were recorded as genotypes with the highest and lowest average anthocyanin contents, respectively (Table 3).

Pantelidis et al*.*^30^ reported that blackberry genotypes have a higher anthocyanin content compared to raspberry and red gooseberry. According to Fan-Chiang and Wrolstad^31^, the anthocyanin content ranged from 70.3 to 201 mg/100 g with an average of 137 mg/100 g among the 51 genotypes of blackberry. Also, Moyer et al.^20^ reported the range of 70 to 201 mg/100 g and an average of 141 mg/100 g in the fruit, as a result of investigating 27 blackberry hybrids. All of those previous reports are in general agreement with the results obtained in this study. Interestingly there was a significant negative association between latitude of the original habitats of the studied *Rubus* genotypes and their fruit anthocyanin (r =  − 0.287). It should be noted that plants sampled in higher latitude evolved in Caspian see which experience rainy and cloudy weather and less light intensity.

#### TSS

Total soluble solids ranged from 7.9°Brix (in the ‘Talesh 2’ genotype) to 17.8°Brix (in the ‘Kazerun’ (Eslamabad) genotype) among the accessions of the four species studied (Table 3). The increase in TSS can be a result of longer photoperiods or higher intensities of light. TSS was probably dependent on temperature and field conditions, as blackberry plants that were located at higher latitudes received less light. For example, blackberries grown in Oregon had relatively higher TSS levels than those grown in Mexico. That is probably because the latter genotypes were adapted to longer photoperiods^27^. In line with mentioned example, considering the geographic parameters of the natural habitats in which studied *Rubus* genotypes collected (Table 5), there were a significant positive correlation between altitude and fruit TSS (r = 0.287), but a negative association between latitude and TSS (r =  − 0.387). Evidently, TSS is a better indicator of fruit maturity in most of the products, especially in blackberry, and has a very important role in the food industry^32^. Previously, Reyes-Carmona et al.^27^ recorded a TSS value of 16.1% in the ‘Evergreen’ cultivar from Woodburn as the highest TSS, and 7.5% in ‘Brazos’ from Ziracuaretiro as the lowest TSS among the wild blackberry genotypes grown in Mexico. Variation in TSS was also reported by Pantelidis et al*.* and Clark and Finn.

#### Total phenol

The fruits’ total phenol content, considering all genotypes of the species investigated, ranged from 109.5 mg/100 g to 129.1 mg/100 g. The genotypes ‘Anzali 2’ (*R. hirtus*) and ‘Sepidan’ (Roodbal) (*R. sanctus*) were considered to have the highest and lowest total phenols, respectively (Table 3). Among all of the evaluated fruit characteristics, the lowest diversity index was attributed to the total phenol content (4.86) which was six times less than the variation recorded for berry weight and four times less than that of the antioxidant activity (Table 4).

Pantelidis et al.^30^ reported the highest total phenol in blackberry compared to other crops such as raspberries, red currants, goose berries and cornelian cherries. According to Gundogdu et al.^11^, the phenolic compounds that were found chiefly in blackberry genotypes were catchin, ranging from 111.599 to 438.970 mg/100 g, and ellagic acid ranging from 10.610 to 51.506 mg/100 g in the fruits of blackberry cultivars from East Anatolia. Berry crops including blackberries are important sources of polyphenolic compounds for the human diet, and they have been examined for phenolic content frequently. Nonetheless, a high level of phenolic content is not a reliable sign of the general quality of products. Accordingly, more comprehensive experimental evidence is needed to confirm the beneficial effects of phenolic compounds in these crops^33^.

#### Titratable acid (TA)

Considering all accessions of the four species, TA content ranged from 0.36 to 0.83%. In this respect, the genotypes ‘Namak Abrud 2’ and ‘Jadeh Kandovan’ (of the *R. sanctus*) were ranked as genotypes with the highest and lowest TA contents (Table 3). The diversity index of TA in this study equaled 20.48 (Table 4). Clearly, the ratio of TSS to TA contribute greatly to the taste of blackberry and it is therefore very important in selecting genotypes for breeding programs^34^. An investigation on the total acid content and malic acid of the five blackberry genotypes revealed that they varied from 1.3 to 25.9 g kg^−1^ and from 0.6 to 11.0 g kg^−1^, respectively^35^. A similar study was carried out on blackberry genotypes in Italy and showed that the fruits’ citric acid content ranged from 1.1 to 16.7 g kg^−1^ and that the malic acid ranged from 4.0 to 15.8 g kg^−1^^13^. Furthermore, Gundogdu et al.^11^ reported the highest citric acid (7.131 g kg^−1^) and succinic acid (2.021 g kg^−1^) in ‘Cherokee’ and ‘Bursa 2’ cultivars. There is a report on wild blackberry that shows the total acidity ranged from 1.02 to 4.22% in the ‘Evergreen’ (from Oregon) and Patzcuaro (from Mexico), respectively^27^. These results are quite higher than those obtained in this study. These differences could be attributed to varied climatic conditions, i.e. higher light intensities in Iran, as well as the different species or genotypes.

#### Ascorbic acid (AA)

Variations in the AA among all genotypes of the four species ranged from 9.56 mg/100 g to 20.92 mg/100 g. The genotypes ‘Sari 3’ and ‘Nourabad (Bavan)’ (of the *R. sanctus*) were recognized as genotypes with the maximum and minimum values of AA, respectively (Table 3). The estimated diversity index for the AA content was 17.68 (Table 4).

Blackberries are among the products with high ascorbic acid contents. They can have an important role in supplying this important vitamin to consumers. Reyes-Carmona et al.^27^ estimated the amount of ascorbic acid in different genotypes and reported that the ascorbic acid ranged from 0.80 meq/g in ‘Comanche’ to 0.82 meq/g in ‘Brazos’. No significant differences were observed in the ascorbic acid content, even in genotypes considered from different origins. Ochmian et al.^36^ reported an ascorbic acid content of 11 mg/100 g in the blackberry. Gundogdu et al.^11^ also recorded ascorbic acid contents between 10.288 and 25.399 mg/100 g among blackberry genotypes that originated from East Anatolia in Turkey, which is in agreement with the findings of the current study.

### Correlation among the measured traits and also between geographic parameters and measured traits

Results of correlation analysis concerning the plant chemical data are presented in Table 6 and Fig. 3. Results showed that the antioxidant activity correlated highly and positively with all of the measured attributes including ascorbic acid (r = 0.927), anthocyanin (r = 0.752), total phenol (r = 0.681), TSS (r = 0.473) and berry weight (r = 0.541). However, the antioxidant activity did not show a significant correlation with titratable acidity. Fruit weight correlated positively with anthocyanin (r = 0.591), ascorbic acid (r = 0.508), total phenol (r = 0.412) and TSS (r = 0.264), but correlated negatively with titratable acidity (r =  − 0.281). The TSS also correlated positively with anthocyanin (r = 0.429), total phenol (r = 0.484) and ascorbic acid (r = 0.342). The values of ascorbic acid and the total phenol correlated positively with each other (r = 0.646).

**Table 6:** Person simple Correlations between berry weight, fruit bio-chemical characteristic and antioxidant activity in 57 blackberry genotypes assigned to four wild species (mean of 2014 and 2015).

	Fruit weight (g)	Antioxidant activity (%)	Anthocyanin (mg/100 g)	TSS (°Brix)	Total phenol (mg/100 g)	Titrateable acid (%)	Ascorbic acid (mg/100 g)
Fruit weight (g)		0.541**	0.591**	0.264**	0.412**	− 0.281**	0.508**
Antioxidant (%)			0.752**	0.473**	0.681**	0.066^ns^	0.927**
Anthocyanin (mg/100 g)				0.429**	0.577**	− 0.124^ns^	0.677**
TSS (°Brix)					0.484**	0.096^ns^	0.342**
Phenol (mg/100 g)						0.096^ns^	0.646**
Titrateable acid (%)							0.026^ns^
Ascorbic acid (mg/100 g)							

* and **Significant different at 5% and 1% probability levels respectively.

According to Fig. 3 which represents the bi-plot of principle component analysis, the cosine of the angles between vectors shows the extent of correlation between traits which is in accordance to that of correlation analysis in almost all of studied traits. The acute angles (< 90°) represent positive correlations, whereas wide obtuse angles (90° <) show a negative correlation. The length of the vectors connecting traits to the origin shows the extent of variability.

Correlations between the bio-chemical characteristic and geographic information (Table 5) showed that, latitude has a significant negative correlation with TSS, anthocyanin and berry weight (− 0.387, − 0.366 and − 0.287, respectively). It worth to mention that the higher latitudes in which many of the genotypes sampled, are located in Caspian Sea region which experience more rain and cloudy weather than other regions. On the other hand, altitude has a positive and significant correlation with fruit TSS (0.287). However, several environmental factors (such as temperatures, light, rainfall) that was effected by altitude, longitude and latitude can influence on photosynthetic rate, fruit size, sugar-to-acid ratio, fruit quality and etc.^7,37^. Association between geographical parameters and some of plant features like leaf and fruit size has been reported previously by Sorkheh et al.^24^ in wild almond species from Iran.

The success of breeding programs is highly dependent on our knowledge of desirable traits and their outcomes through crosses. Established relationships between desirable traits can help breeders with parental partner selection in breeding programs. Correlations between traits indicate whether the selection of one trait has an effect on another. Strong correlations between traits could help breeders select the important traits indirectly. This can accelerate and facilitate breeding programs. Furthermore, a close relationship between traits may also facilitate or hinder gene introgression since the selection of a trait could favor the presence of another desirable trait.

Wang and Lin^38^ found a linear correlation between total antioxidant capacity and phenol content in both blackberries (r = 0.961) and raspberries (r = 0.911). Deighton et al.^39^ reported that there was an apparent linear relationship between antioxidant capacity and total phenols (r = 0.965), whereas anthocyanin content had a minor influence on antioxidant activity (r = 0.588) and ascorbic acid contributed only minimally to the antioxidant potential of *Rubus* juices. Correlations obtained between the chemicals in this study are in general agreement with the findings reported by Yilmaz et al.^6^, Guerrero et al.^21^, Huang et al.^29^, Maro et al.^12^ and Gharaghani et al.^16^. However, Rekika et al.^40^ did not find correlations between anthocyanin contents, antioxidant capacity and ellagic acid.

### Cluster analysis

**Figure 2:**
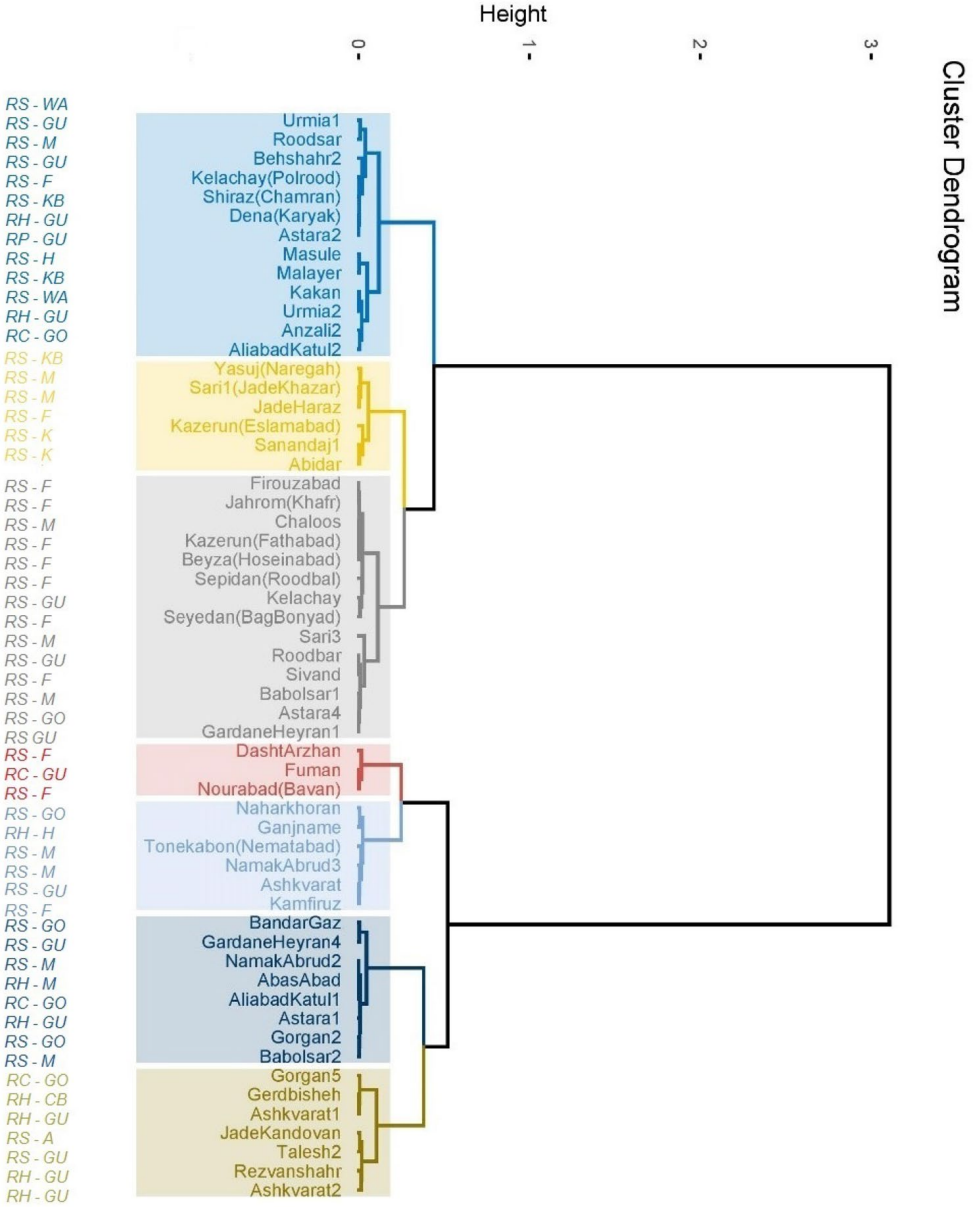
Dendrogram for clustering 57 blackberry genotypes assigned to four wild species based on all evaluated traits (left column shows the abbreviations for the species and provinces (RS, RP, RH and RC represents *Rubus sanctus*, *R. persicus*, *R. hirtus* and *R. caesius*, respectively. A, M, K, F, H, CB, WA, KB, GU and GO represents Alborz, Mazandaran, Kurdistan, Fars, Hamadan, Charmahal-Bakhtiari, West Azarbayejan, Kohgiluyeh-Boyerahmad, Guilan and Golestan provinces, respectively).

Cluster analysis of accessions was performed by considering all of the measured attributes (Fig. 2). According to the dendrogram, the genotypes being studied were divided into two main groups. The first cluster contains the majority of *R. sanctus* accessions, the only accession of *R. persicus* (‘Masule’), one accession of *R. caesius* (‘Aliabad Katul 2’) and two of *R. hirtus* accessions (‘Astara 2’ and ‘Anzali 2’). Cluster 2 was comprised of the other three accessions of *R. caesius* (‘Aliabad Katul 2’, ‘Fuman’ and ‘Gorgan 5’), all of the *R. hirtus* accessions (except two mentioned before) as well as some accessions of *R. sanctus* (most of which were from the Caspian Sea region in Golestan, Mazandaran and Guilan provinces, with a few exceptions from Fars provinces).

In general, the results of cluster analysis showed a partial differentiation between the genotypes based on their species and, to lesser extent, according to their origin within the main clusters (Fig. 2). For example, ‘Ali Abad Katul 2’ of *R. caesius* and two of *R. hirtus* accessions were positioned among *R. sanctus* accessions in cluster 1and a minority of *R. sanctus* genotypes were clustered with accessions from other species in second clade. However, the genotypes from different origins were grouped together in some cases, but there were also many cases that genotypes of close regions placed in same cluster or sub-clusters. For example, 8 out of 12 accessions of *R. sanctus* from Fars province were placed in third sub-cluster of the first cluster. Two accessions of *R. sanctus* from West Azarbayejan were located in first sub-cluster and two others from Kurdistan were positioned in second sub-cluster of the first clade.

The genetic material of plants could have been exchanged between the regions studied herein, and this can partly explain the reason why the genotypes of different geographic origins could be grouped together. Nonetheless, genotypes of the same geographic origin may not necessarily be positioned in the same cluster. However, it should be mentioned that this analysis is based on a limited number of bio-chemical traits and their corresponding data. Therefore, it may not be as reliable as larger numbers of morpho-chemical traits or molecular data. Gharaghani et al.^16^ studied the genetic diversity of *R. sanctus* genotypes growing in two distinct climatic conditions including the Caspian Sea region which is situated in north of Iran, and in the southern regions of Zagros being located southwest of Iran. According to that study, 16 blackberry genotypes were grouped into two main clusters regardless of their origins. It should however be mentioned that they investigated the genotypes by collecting their fruits from plants as they were situated in their respective places of origin, and more of the fruit attributes were measured, including physical and chemical traits. However, the current research investigated the plants as they were transferred and established in a repository, i.e. all in the same environment. Badjakov et al*.*^14^ analyzed 28 raspberry genotypes including 18 Bulgarian genotypes and breeding lines, 8 accessions from outside Bulgaria and two accessions of wild species, including *R. occidentalis* and *R. adiene*. Their genotypes were clearly clustered into two groups corresponding with two pedigree groups. Guerrero et al.^21^ used similar methods of cluster analysis to group genotypes and cultivars of wild and cultivated berries including blueberry, raspberry, Chilean guava and red sarsaparilla, based on their antioxidant capacity in Chile.

#### Principle component analysis (PCA)

**Figure 3:**
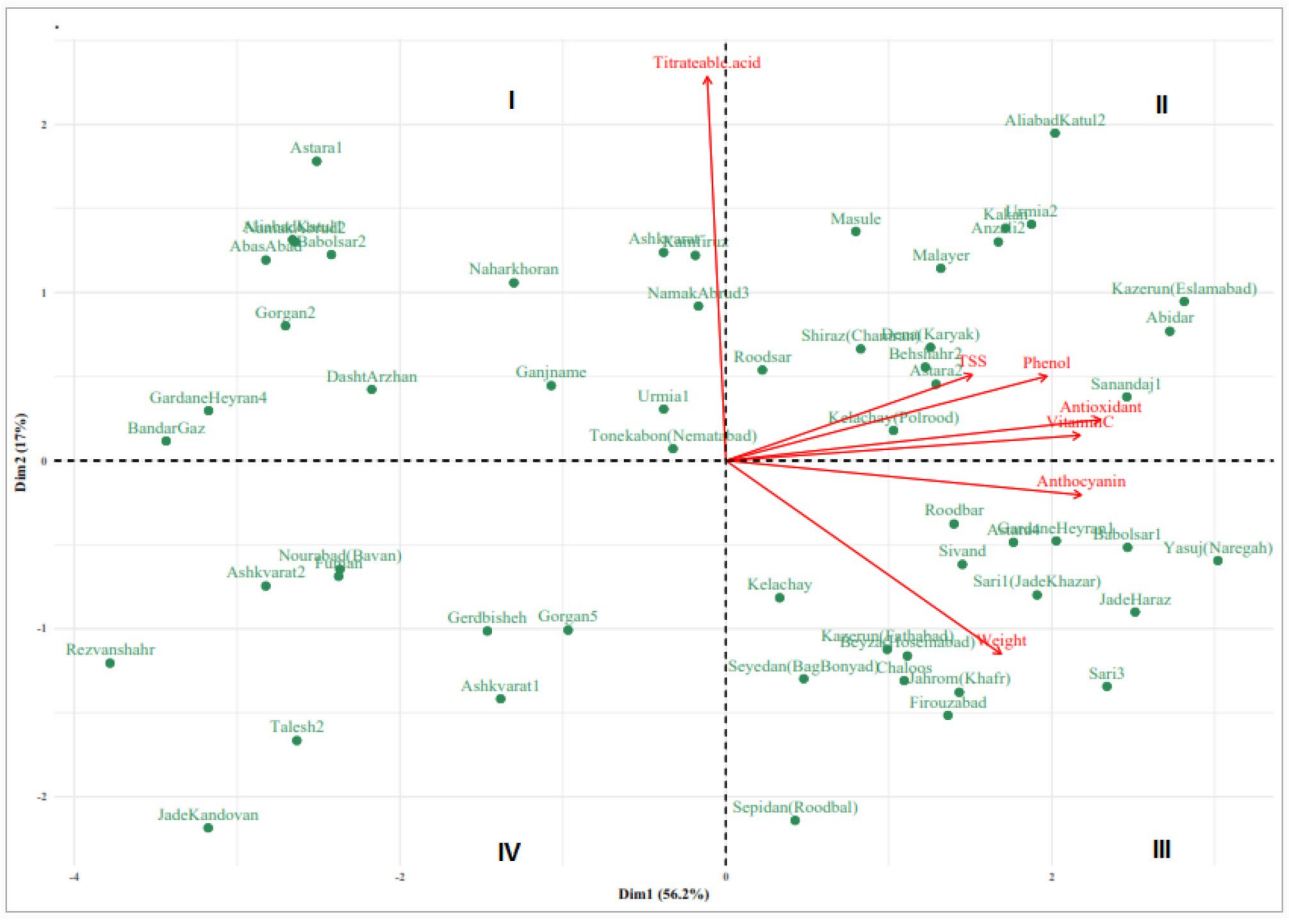
Projection of studied blackberry accessions based on the first (PCA1) and second (PCA2) principal components and vectors of studied traits.

In PCA analysis, the first two PCAs determined 73.2% of the total variations of accessions in studied traits. The first PCA (PCA1) that was associated with TSS, total phenol, antioxidant capacity, vitamin C, anthocyanin and berry weight accounted for 56.2% of the variations, whereas, the second PCA (PCA2) with 17.0% share in the total variation was associated with titrateable acidity and to some extent berry weight (Fig. 3).

Based on the bi-plot of the first two PCAs, genotypes and traits classified into four quadrants. The projection of genotypes on the bi-plot showed that genotypes placed in the quadrant I had higher titrateable acidity. Accordingly, the genotypes scattered in the quadrant II had higher TSS, total phenol, antioxidant activity and vitamin C. The genotypes positioned in quadrant III represent high berry weight and anthocyanin content. Accessions in quadrants IV had lower quantity in almost all of studied traits (Fig. 3). This grouping is in agreement with that of cluster analysis, considering the fact that almost all of accessions in quadrants II and III (with a few exceptions) were positioned in the first clade of cluster analysis and the genotypes grouped in quadrants I and IV, represents the accessions of clade 2 in cluster analysis.

According to Fig. 3, the cosine of the angles between vectors shows the extent of correlation between traits. The acute angles (< 90°) represent positive correlations, whereas wide obtuse angles (90° <) show a negative correlation. For example, wide angles between vector for titrateable acidity and those of other trait shows the weak association of these traits, this is while, the very narrow angle between antioxidant activity and vitamin C content shows the high correlation of these attributes. These results are fully in accordance to that of correlation analysis in almost all of studied traits (Table 5). The PCA is a multivariate statistical analysis which can be used to determine the number of main factors with the purpose of reducing the number of effective parameters to discriminate genotypes^27,41^. Previously, PCA had been used to establish genetic relationships among cultivars and genotypes as well as to study the correlations among plant biochemical traits of different *Rubus* species in Iran^38^ and some other countries^6,42^.

## Conclusion

Results showed that a vast majority of genetic diversity is available for berry weight, fruit bio-chemical traits and antioxidant activity in the Iranian wild *Rubus* species. This diversity has so far remained untapped for the development of new cultivars in breeding programs. Despite the profitable traits, however, some traits like the spiny bush trait often exist in the superior genotypes which make them less likely to be used directly as commercial cultivars. Strong correlations were detected among the traits being studied, especially between the anti-oxidant capacity and other measured chemicals like anthocyanin and total phenols, which could help breeders select these traits indirectly. This could facilitate and accelerate breeding programs. Cluster analysis showed a relatively good discrimination between the genotypes based on their species and, to some extent, according to their origin. However, in some cases, genotypes were grouped together even though they were from different origins. Moreover, it should be mentioned that this analysis is based on limited numbers of bio-chemical traits and their data. Further studies based on larger numbers of morpho-chemical traits or molecular data are needed to shed a stronger light on the relationships among the species and genotypes under consideration.
